# Long-term treatment with acylated analogues of apelin-13 amide ameliorates diabetes and improves lipid profile of high-fat fed mice

**DOI:** 10.1371/journal.pone.0202350

**Published:** 2018-08-29

**Authors:** Finbarr P. M. O’Harte, Vadivel Parthsarathy, Christopher Hogg, Peter R. Flatt

**Affiliations:** School of Biomedical Sciences, SAAD Centre for Pharmacy and Diabetes, Ulster University, Coleraine, Co. Londonderry, Northern Ireland, United Kingdom; University of Lancaster, UNITED KINGDOM

## Abstract

Previous studies have shown that modified apelin analogues exhibited enzyme resistance in plasma and improved circulating half-life compared to apelin-13. This study investigated the antidiabetic effects of chronic administration of stable long acting fatty acid modified apelin analogues, namely, (Lys^8^GluPAL)apelin-13 amide and pGlu(Lys^8^GluPAL)apelin-13 amide, in high-fat fed obese-diabetic mice. Male NIH Swiss mice (groups n = 8) were maintained either on a high-fat diet (45% fat) from 8 to 28 weeks old, or control mice were fed a normal diet (10% fat). When diet induced obesity-diabetes was established after high-fat feeding, mice were injected i.p. once daily with apelin analogues, liraglutide (25 nmol/kg) or saline (controls). Administration of (Lys^8^GluPAL)apelin-13 amide and pGlu(Lys^8^GluPAL)apelin-13 amide for 28 days significantly reduced food intake and decreased body weight. Non-fasting glucose was reduced (p<0.01 to p<0.001) and plasma insulin concentrations increased (p<0.01 to p<0.001). This was accompanied by enhanced insulin responses (p<0.01 to p<0.001) and significant reductions in glucose excursion after oral (p<0.01) or i.p. (p<0.01) glucose challenges and feeding. Apelin analogues also significantly improved HbA_1c_ (p<0.01), enhanced insulin sensitivity (p<0.01), reduced triglycerides (p<0.001), increased HDL-cholesterol (p<0.01) and decreased LDL-cholesterol (p<0.01), compared to high-fat fed saline treated control mice. Cholesterol levels were decreased (p<0.01) by pGlu(Lys^8^GluPAL)apelin-13 amide and both apelin treated groups showed improved bone mineral content, reduced fat deposits and increased plasma GLP-1. Daily treatment with liraglutide mirrored many of these changes (not on bone or adipose tissue), but unlike apelin analogues increased plasma amylase. Consumption of O_2_, production of CO_2_, respiratory exchange ratio and energy expenditure were improved by apelin analogues. These results indicate that long-term treatment with acylated analogues (Lys^8^GluPAL)apelin-13 amide and particularly pGlu(Lys^8^GluPAL)apelin-13 amide resulted in similar or enhanced therapeutic responses to liraglutide in high-fat fed mice. Fatty acid derived apelin analogues represent a new and exciting development in the treatment of obesity-diabetes.

## 1. Introduction

Type 2 diabetes mellitus (T2DM) is characterised by defective regulation of carbohydrate, lipid and protein metabolism [1]. The biochemical hallmark of T2DM is chronic hyperglycemia resulting from defects in insulin secretion and action [2,3]. More than 425 million people worldwide are affected by the disease which will increase to 628 million by 2045 [4]. This increase is due to improved life expectancy, obesity and an increase in the populations of ethnic groups at higher risk of the disease [5]. For example in the UK there is an increased predisposition of disease in some ethnic populations (e.g. South Asians, Indian and Pakistani) and this could be due to dietary nutritional deficiencies and or reduced levels of habitual physical activity [6,7]. Uncontrolled hyperglycemia leads to macrovascular and microvascular complications such as cardiovascular disease [8] retinopathy, neuropathy and nephropathy [9,10,11]. Persistent lifestyle changes and pharmacological intervention are essential to achieve good metabolic control and reduce the risk of hyperglycemia induced complications.

Visceral obesity is the most common risk factor associated with of T2DM and other chronic diseases, including atherosclerosis, arterial hypertension [12]. The modern Western diet coupled with a sedentary lifestyle has led to a pandemic of obesity which is now a severe public health issue [13,14]. Pharmaceutical agents with dual actions to correct hyperglycemia and promote weight are few and far between with only GLP-1 receptor agonists proving to be effective [15,16]. Even then, side-effects such as nausea, pancreatitis and possible cancer risk limit their usefulness [17,18,19]. Thus there is a significant need for development of new multi-faceted pharmaceutical agents, which induce weight loss and decrease both hyperglycemia and associated complications without causing adverse effects.

Apelin is an adipokine and circulating peptide produced and secreted by adipocytes [20]. Several bioactive apelin peptides, including apelin-12, -13, -16, -17, -19 and -36 are products of APLN gene, located on chromosome 11q12 [21] with apelin-13 and apelin-36 being the most abundant and biologically active forms [22]. The human apelin receptor, APJ is ubiquitously present in tissues [21,23] and the apelinergic system has been shown to be involved in multiple metabolic processes including control of glucose homeostasis [24,25,26].

Rapid degradation and short half-life of native apelin isoforms (4–7 min) severely hinders the pharmacological exploitation of apelin peptides [27]. To overcome this problem, we have developed enzyme resistant fatty acid derived analogues of apelin-13 namely (Lys^8^GluPAL)apelin-13 amide and pGlu(Lys^8^GluPAL)apelin-13 amide [28]. Notably, these analogues have the identical fatty acid moiety conjugated to Lys^8^ using the same chemical linker as the GLP-1 mimetic, liraglutide [29]. These stable apelin analogues stimulated insulin secretion from clonal pancreatic beta cells, primary culture of isolated mouse islets cells being the most potent of a series of analogues studied [30]. In the present study, metabolic and weight reducing effects of chronic once daily administration of (Lys^8^GluPAL)apelin-13 amide and pGlu(Lys^8^GluPAL)apelin-13 amide were directly compared to the GLP-1 mimetic, liraglutide using a high-fat fed mouse model diet-induced obesity-diabetes (DIO).

## 2. Materials & methods

### 2.1. Peptides

All apelin analogues used in the study were custom made by EZ Biolabs (Carmel, IN, USA) at >95% purity. Purity of the peptides was checked by RP-HPLC and structural identity confirmed by electrospray ionization mass spectrometry as described previously [28]. Briefly, modifications of native apelin-13 peptide were carried out to confer enzyme resistance to prolong the biological activity. Furthermore, a gamma-glutamyl spacer with palmitate adjunct (GluPAL) was added to the side-chain of apelin Lys^8^ to promote binding to plasma proteins and reduce renal clearance, thus extending the *in vivo* bioactivity. These peptides are only known to bind to and activate the APJ receptor and the half-life *in vitro* was >24 h [28].

### 2.2. Experimental animals

Male NIH Swiss mice (Harlan UK Ltd., Blackthorne, UK) were housed individually in an air-conditioned room (22 ± 2°C) with relative humidity of 51 ± 5% and a 12 h light: dark cycle (08:00–20:00 h). Drinking water was freely available. Animals were maintained on a high fat diet (45% fat, 20% protein, 35% carbohydrate; percent of total energy 26.15 kJ/g; Dietex International Ltd., Witham, UK) from 8 weeks of age for a total of 150 days to evoke dietary-induced obesity-diabetes (DIO). Another group of mice was maintained on standard rodent diet (10% fat, 30% protein, 60% carbohydrate; percent of total energy 12.99 kJ/g, Trouw Nutrition, Cheshire, UK) and used as a model of normal controls. Similar high-fat diets, containing a large percentage of energy from fat, are used routinely in obesity-diabetes research [31–33].

### 2.3. Chronic treatment and metabolic effects

Groups of normal control and high-fat fed mice (n = 8) received once daily intraperitoneal injections of either 0.9% saline vehicle (lean and high fat controls) or either (Lys^8^GluPAL)apelin-13 amide, pGlu(Lys^8^GluPAL)apelin-13 amide or liraglutide (each at 25 nmol/kg bw) over a 28 day treatment period. Food intake, bodyweight, non-fasting blood glucose and plasma insulin concentrations were measured every 2–3 days. Following the 28 days, 16 h fasted mice were administered with glucose (18 nmol/kg body weight) either intraperitoneally or orally. To measure the insulin sensitivity, hypoglycemic response was measured following administration of insulin (25 U/kg). Blood samples were collected from cut tail tips of mice and blood glucose (Ascencia Contour meter) and HbA_1c_ (PTS Diagnostic, IN, USA) were measured. Blood was collected into chilled fluoride/heparin-coated microcentrifuge tubes (Sarstedt, Nümbrecht, Germany) and centrifuged (13,000g × 3 min) using a Beckman microcentrifuge (Beckman Instruments, Palo Alto, CA, USA). The resulting plasma was then aliquoted into fresh Eppendorf tubes and stored at −20°C for subsequent biochemical analysis. Fat mass, bone mineral content (BMC) and bone mineral density (BMD) were assessed using the PIXImus DEXA scanner. Measurements of indirect calorimetry, energy expenditure and locomotor activity were assessed using comprehensive laboratory animal monitoring system (CLAMS) metabolic chambers as described previously [34]. Although the main intervention study was carried out for a period of 28 days, the various peptide treatments were extended beyond that period to allow for all of the additional investigations to be performed while maintaining the once daily peptide treatment regime. Thus, all post-intervention experiments were performed between day 28 and day 40. On day 28 body weight, glucose and insulin measurements were carried out. The glucose tolerance test (GTT) and other terminal testing like glycated haemoglobin HbA_1c_, blood biomarkers were conducted between day 28 and 35. The CLAMS analysis was performed between days 36 and 38 where mice were give 24 h to acclimatise and a further 24 h for measurements, as described previously [35]. Terminal blood samples and tissue retrieval was completed on day 40.

### 2.5. Terminal analysis

At the end of the experimental period (day 40), pancreatic tissues were excised for analysis of insulin content [36,37]. Blood was taken from fasted mice for measurement of lipid profiles including HDL-cholesterol, LDL-cholesterol and triglyceride levels by an ILab 650 Clinical Analyser (Instrumentation Laboratory, Warrington, UK). Amylase activity (Amylase assay kit, Abcam, UK) and circulating total GLP-1 (ELISA, Millipore, UK) concentrations was measured as described in manufacturer’s protocol [38].

### 2.6 Ethical standard

All animal experiments were carried out in accordance with the UK Animals (Scientific Procedures) Act 1986 and EU Directive 2010/63EU for animal experiments and approved by Ulster University Animal Ethics Review Committee. All necessary steps were taken to prevent any potential animal suffering.

### 2.7. Statistical analysis

All data was analysed with Prism (v.5.0, GraphPad Software Inc. CA, USA) and expressed as mean ± S.E.M. Bodyweight, glucose, insulin and all GTT data were analysed using two-way analysis of variance (ANOVA) followed by the student-Newman-Keuls *post-hoc* test. Cumulative food intake was analysed using Student’s t-test. Area under the curve (AUC) was calculated using trapezoidal rule with baseline correction. All other data including AUC were analysed using one-way ANOVA. p<0.05 was considered to be statistically significant.

## 3. Results

### 3.1. Chronic administration of acylated apelin-13 amide analogues improves metabolic status in high-fat fed mice

**Fig 1:**
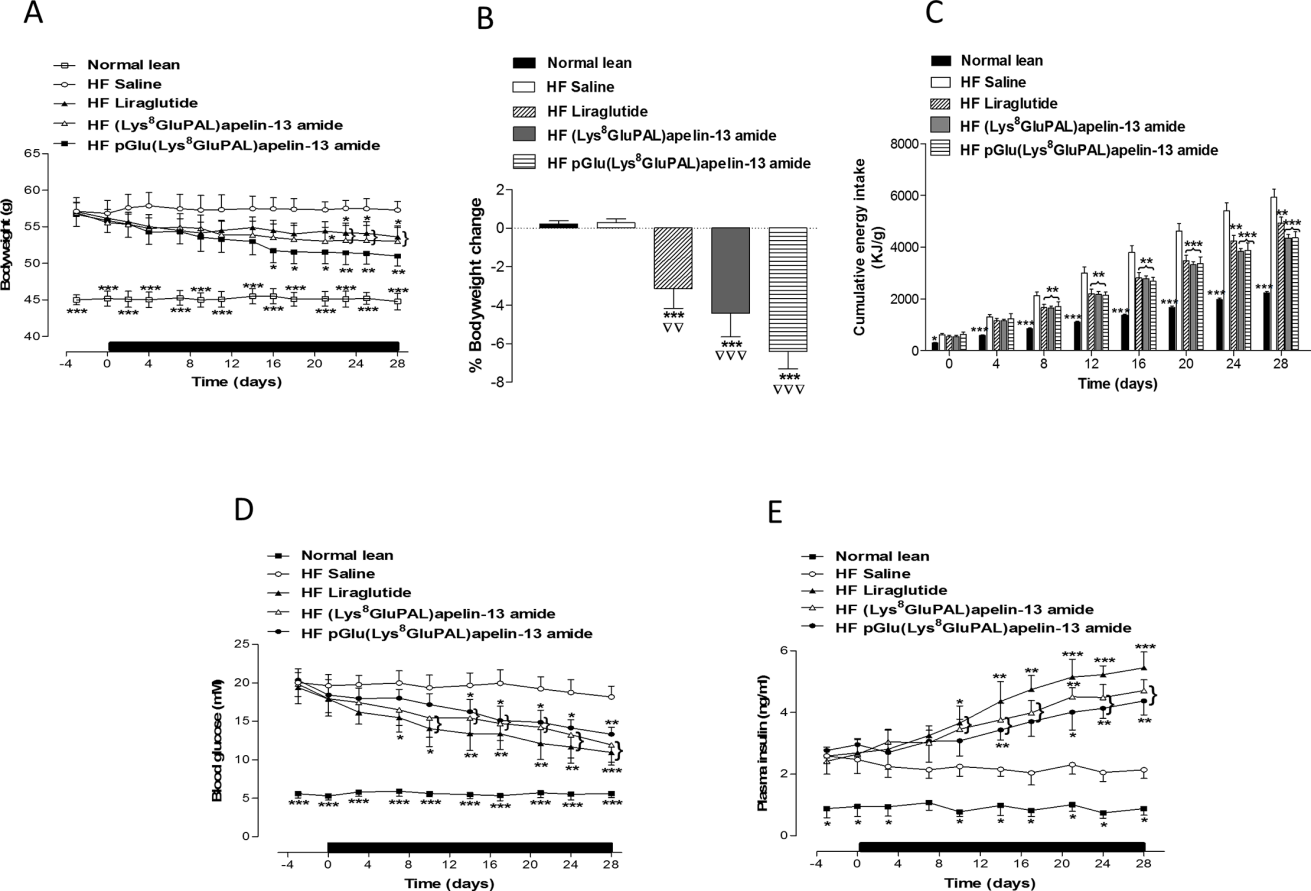
Chronic effect of once daily i.p. administration of liraglutide, (Lys^8^GluPAL)apelin-13 amide or pGlu(Lys^8^GluPAL)apelin-13 amide (each at 25 nmol/kg bw) for 28 days on body weight (A), % weight change (B), cumulative energy intake (C), non fasting blood glucose (D) and plasma insulin (E). Treatment period represented by black horizontal bar. Values represent mean ± S.E.M. (n = 8) where *p<0.05, **p<0.01 and ***P<0.001 is compared to high-fat fed saline treated mice, ^**▽▽**^p<0.01 and ^**▽▽▽**^p<0.001 is compared to lean mice fed on a normal diet.

A significant decrease in % bodyweight change was noted with all treatment groups compared to lean and high-fat fed saline treated mice (p<0.01 and p<0.001; Fig 1A and 1B). Cumulative energy intake was also significantly decreased in apelin treated mice (27% decrease, P<0.01; Fig 1C). Both acylated apelin-13 amide analogues significantly decreased non-fasted blood glucose (P<0.05 and P<0.01; Fig 1D) and increased non-fasting plasma insulin (P<0.05 and P<0.01; Fig 1E). Liraglutide evoked similar effects although, with the exception of plasma insulin, with latter onset or less durability.

### 3.2. Chronic administration of acylated apelin-13 amide analogues reduces glycemic excursion in high-fat fed mice

**Fig 2:**
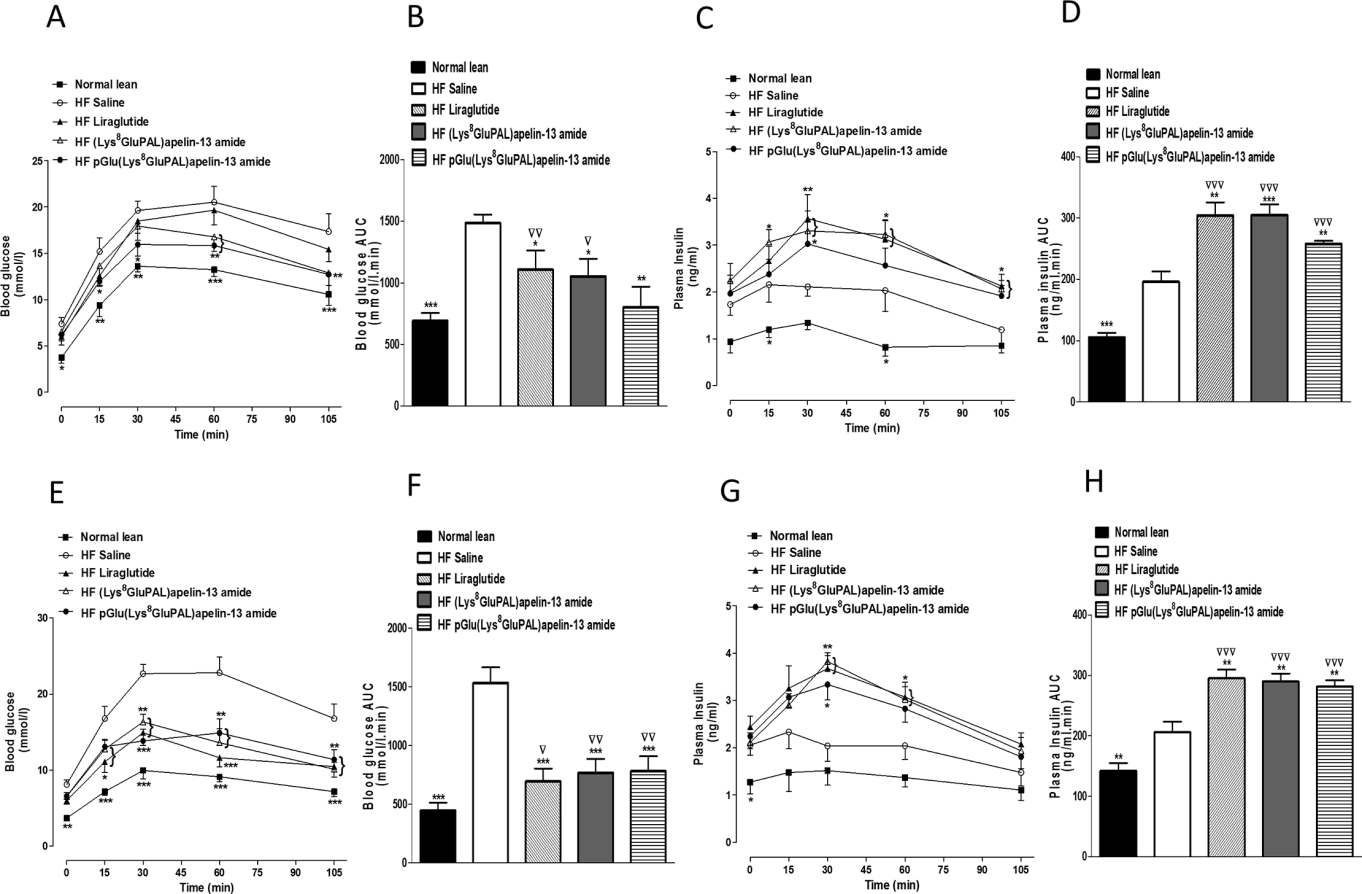
Effect of once daily i.p. administration of liraglutide, (Lys^8^GluPAL)apelin-13 amide or pGlu(Lys^8^GluPAL)apelin-13 amide (each at 25 nmol/kg) on blood glucose and plasma insulin responses to an intraperitoneal (A-D) or oral (E-H) glucose challenge in 18 h fasted high-fat fed mice after 18 hours of fasting. After 28 days, blood glucose (A and E) and plasma insulin concentrations (C and G) were measured before and after administration of glucose (18 mmol/kg body weight). Blood glucose and integrated plasma insulin responses (area under the curve; AUC, 0–105 min) are also shown. Values represent mean ± S.E.M. (n = 8) where *p<0.05, **p<0.01 and ***P<0.001 is compared to high-fat fed saline treated mice, ^**▽**^p<0.05, ^**▽▽**^p<0.01 and ^**▽▽▽**^p<0.001 is compared to lean mice fed on a normal diet.

**Fig 3:**
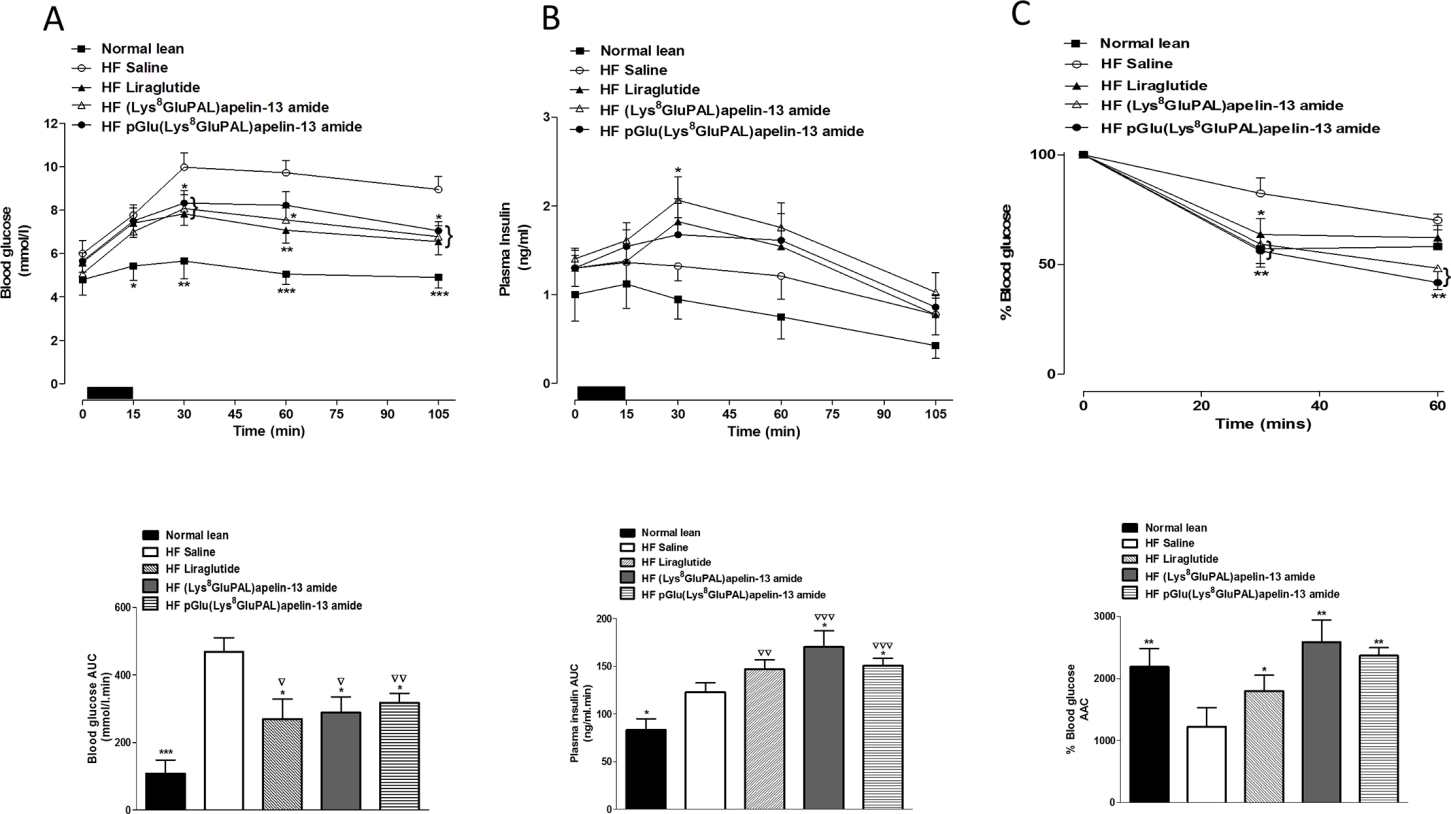
Effect of once daily i.p. administration of liraglutide, (Lys^8^GluPAL)apelin-13 amide or pGlu(Lys^8^GluPAL)apelin-13 amide (each at 25 nmol/kg) on blood glucose (A) and plasma insulin (B) responses to 15 min feeding (A, B) and insulin sensitivity (C) in high-fat fed mice. Tests were performed after 28-days. For meal tests, mice were fasted for 18 h previously and given free access to normal diet for 15 min. The period of feeding is represented by the black horizontal bar. Blood glucose and plasma insulin (AUC) values are also included. For insulin sensitivity tests, insulin (25 U/kg body weight) was administrated by i.p. injection in the fed state. The % blood glucose and AAC values (C) for 0–60 min post-injection are shown. Values represent the mean ± S.E.M. (n = 8) where *p<0.05, **p<0.01 and ***p<0.001 is compared to high-fat fed saline treated mice, ^**▽**^p<0.05, ^**▽▽**^p<0.01 and ^**▽▽▽**^p<0.001 compared to normal mice.

Administration of acylated apelin-13 amide analogues or liraglutide for 28 days, significantly reduced glycemic excursion after an intraperitoneal or oral glucose load (P<0.05 to P<0.001; Fig 2A, 2C, 2E and 2F) and post oral glucose load (P<0.05 to P<0.001; Fig 2E and 2F). pGlu(Lys^8^GluPAL)apelin-13 amide was the most effective as treated mice showed no significant difference in overall glucose excursion compared to lean control mice (Fig 2B). The peptide treatments also significantly increased the overall plasma insulin response after intraperitoneal (P<0.01 and P<0.001; Fig 2C and 2D) or oral glucose load (P<0.05 and P<0.01; Fig 2G and 2H). The insulinotropic responses were much greater than in mice fed on a normal diet (P<0.001, Fig 2D and 2H). Similarly, both acylated apelin-13 amide analogues and liraglutide significantly reduced the blood glucose excursion after 15 min feeding (P<0.05 and P<0.01; Fig 3A). The two acylated apelin-13 amide analogues but not liraglutide significantly increased the plasma insulin response to feeding (P<0.05; Fig 3B). The mean food intake was 0.62, 0.55 and 0.45 g with (Lys^8^GluPAL)apelin-13 amide, pGlu(Lys^8^GluPAL)apelin-13 amide or liraglutide groups, respectively. Insulin sensitivity in all three treatment groups of mice was improved compared with high-fat fed controls (P<0.05 and P<0.01; Fig 3C).

### 3.3. Chronic administration of acylated apelin-13 amide analogues improves terminal biomarkers in high-fat fed mice

**Fig 4:**
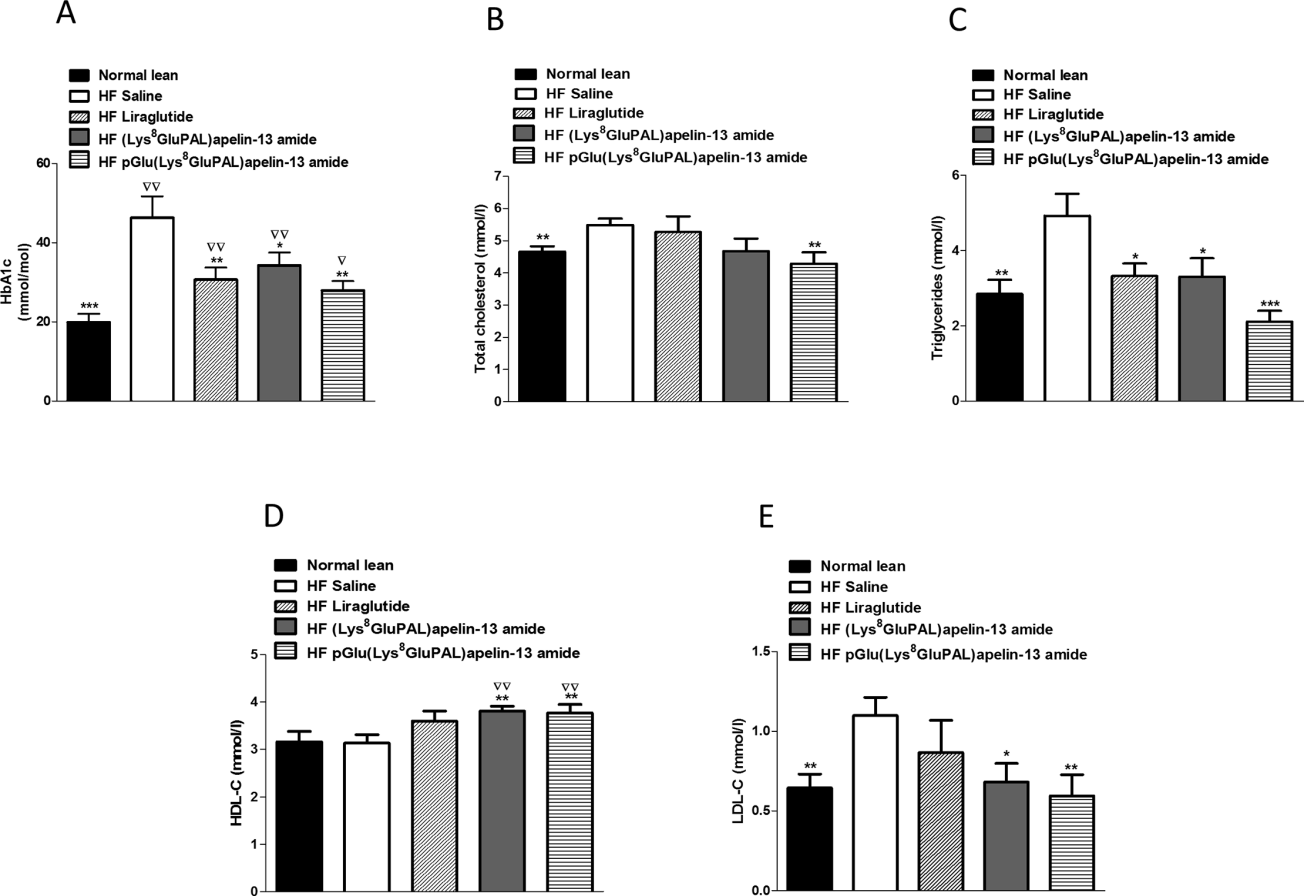
Effect of once daily i.p. administration of liraglutide, (Lys^8^GluPAL)apelin-13 amide or pGlu(Lys^8^GluPAL)apelin-13 amide (each at 25 nmol/kg bw). After 40 days HbA_1c_ (A), plasma total cholesterol (B), triglycerides (C), HDL cholesterol (D) and LDL cholesterol (E), after 40 days of treatment were measured in high-fat fed mice. Values represent the mean ± S.E.M. (n = 8) where *p<0.05, **p<0.01 and ***p<0.001 is compared to high-fat fed saline treated mice, ^**▽**^p<0.05 and ^**▽▽**^p<0.01 is compared to normal mice.

Both acylated apelin-13 amide analogues and liraglutide produced a significant reduction in HbA_1c_ concentration (P<0.05 to P<0.01; Fig 4A). Furthermore, liraglutide (P<0.05), (Lys^8^GluPAL)apelin-13 amide (P<0.05), pGlu(Lys^8^GluPAL)apelin-13 amide (P<0.001) and liraglutide (P<0.05), all significantly reduced circulating triglyceride concentrations (Fig 4C), with only pGlu(Lys^8^GluPAL)apelin-13 amide reducing total cholesterol (P<0.01; Fig 4B). Both acylated apelin-13 amide analogues (P<0.01) significantly increased circulating HDL-cholesterol (Fig 4D) compared to either lean or saline-treated high-fat fed mice. In addition, treatment with (Lys^8^GluPAL)apelin-13 amide (P<0.05) or pGlu(Lys^8^GluPAL)apelin-13 amide (P<0.01) significantly reduced LDL-cholesterol compared to high-fat fed controls (Fig 4E). Such actions were absent from liraglutide treated mice.

### 3.4 Chronic administration of acylated apelin-13 amide analogues increases total GLP-1, bone mineral content and reduces fat mass

**Fig 5:**
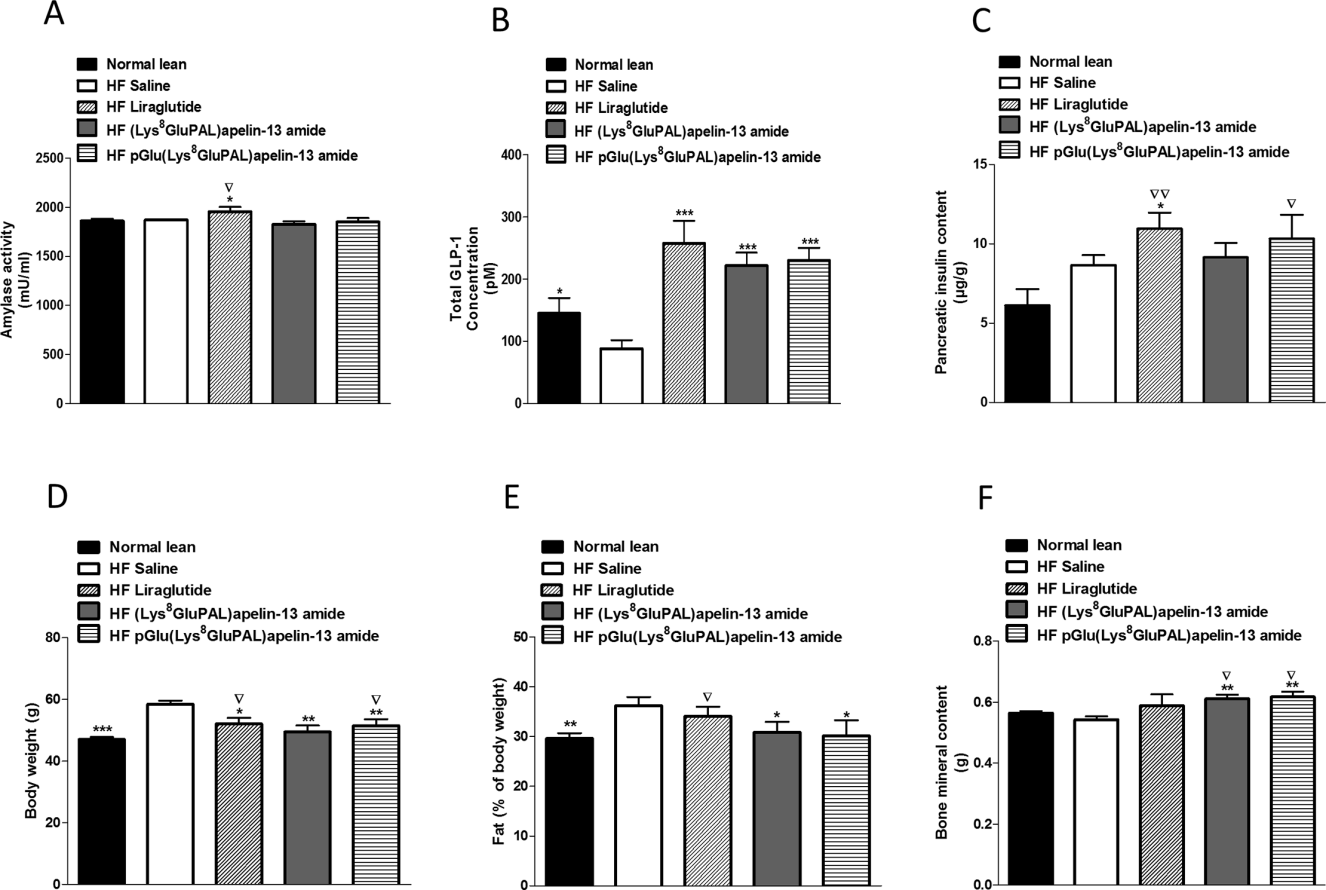
Effect of once daily i.p. administration of liraglutide, (Lys^8^GluPAL)apelin-13 amide or pGlu(Lys^8^GluPAL)apelin-13 amide (each at 25 nmol/kg bw) on α-amylase activity (A), plasma GLP-1 (B), pancreatic insulin content (C), body weight (D), fat mass (%) (E) and bone mineral content (F). Observations were made after 40 days of treatment of high-fat fed and lean control mice. Values represent the mean ± S.E.M. (n = 8) where *p<0.05, **p<0.01 and ***p<0.001 is compared to high-fat fed saline treated mice, ^**▽**^p<0.05 and ^**▽▽**^p<0.01 is compared to normal mice.

Chronic administration of liraglutide significantly increased circulating α-amylase compared to both saline and normal lean mice (P<0.05; Fig 5A). The two acylated apelin-13 amide analogues had no significant effect on this marker of pancreatitis. All treated groups showed enhanced total plasma GLP-1 concentrations (151% - 192%, P<0.001; Fig 5B). Liraglutide was the only treatment to increase pancreatic insulin content (P<0.05; Fig 5C). However, both liraglutide (P<0.01) and pGlu(Lys^8^GluPAL)apelin-13 amide (P<0.05) treated mice developed significantly greater pancreatic insulin stores compared to lean mice. The percentage fat mass was significantly decreased in apelin treated groups compared to high-fat fed controls (P<0.05; Fig 5E). Similarly, both apelin analogues significantly increased bone mineral content (BMC) compared to both high-fat fed controls (P<0.01; Fig 5F), as well as lean mice (P<0.05; Fig 5F).

### 3.5 Chronic administration of acylated apelin-13 amide analogues improves indirect calorimetry, energy expenditure, locomotor activity and feeding bouts in high-fat fed mice

**Fig 6:**
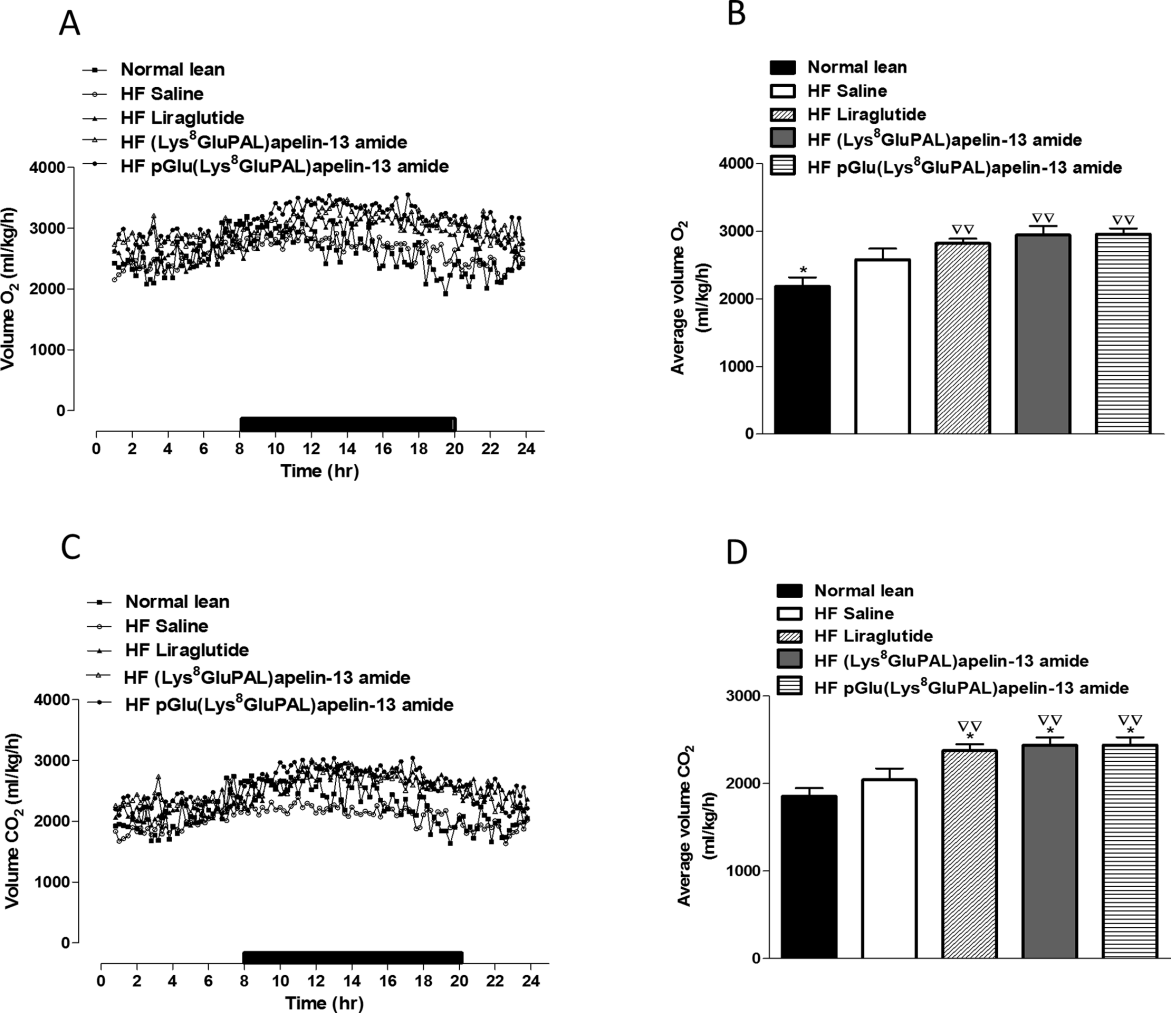
Effect of once daily i.p. administration of liraglutide, (Lys^8^GluPAL)apelin-13 amide or pGlu(Lys^8^GluPAL)apelin-13 amide (each at 25 nmol/kg bw) on O_2_ consumption (A, B) and CO_2_ production (C, D). Following 36-days of treatment mice were placed in CLAMS metabolic chambers for 24 h to acclimatise and a further 24 h for measurements (12 h dark period as shown by the black bar), O_2_ consumption and CO_2_ production were measured for 30 sec at 25 min intervals. Values represent the mean ± S.E.M. (n = 6) where *p<0.01 is compared to high-fat fed saline treated mice and ^**▽▽**^p<0.01 is compared to normal mice.

**Fig 7:**
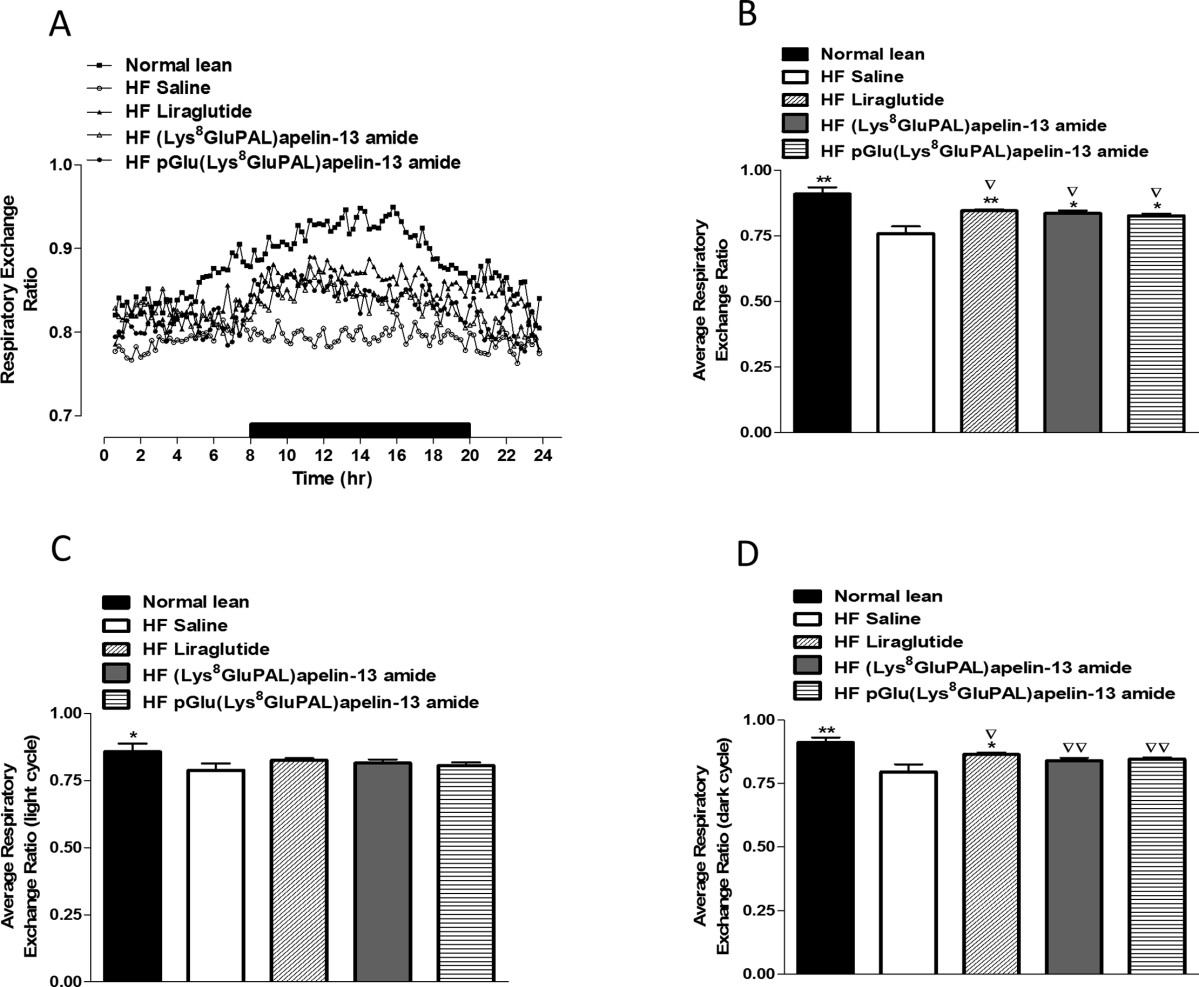
Effect of once daily i.p. administration of liraglutide, (Lys^8^GluPAL)apelin-13 amide or pGlu(Lys^8^GluPAL)apelin-13 amide (each at 25 nmol/kg bw) on respiratory exchange ratio (RER, A). Following 35 days treatment mice were placed in CLAMS metabolic chambers for 24 h (12 h dark period as shown by the black bar). RER was calculated by dividing VCO_2_ by VO_2_. Average RER (B), RER in the light (C) and dark cycles (D) are also included. Values represent the mean ± S.E.M. (n = 6) where *p<0.05 and **p<0.01 is compared to high-fat fed saline treated mice and ^**▽**^p<0.05 and ^**▽▽**^p<0.01 is compared to normal mice.

**Fig 8:**
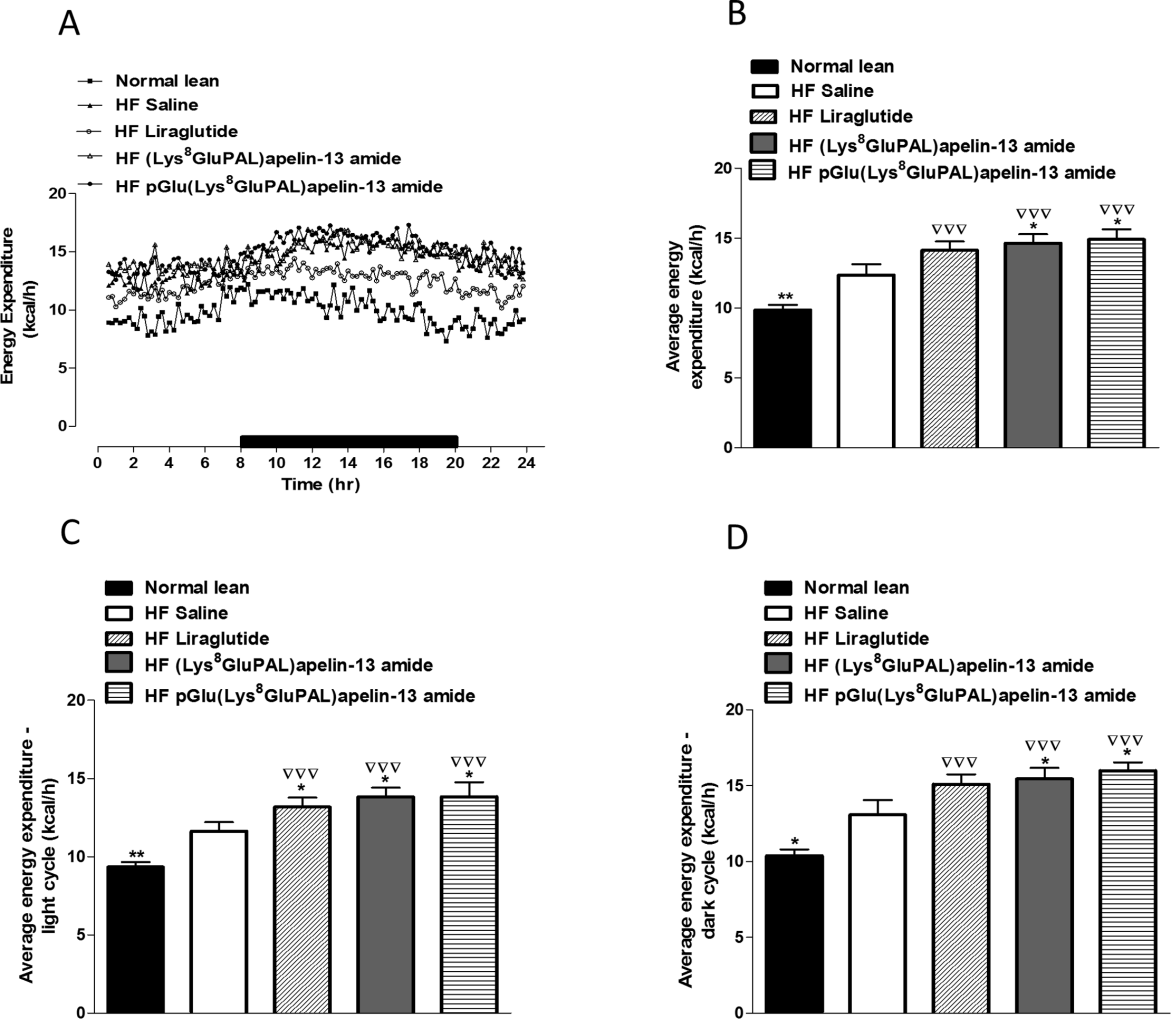
Effect of once daily i.p. administration of liraglutide, (Lys^8^GluPAL)apelin-13 amide or pGlu(Lys^8^GluPAL)apelin-13 amide (each at 25 nmol/kg bw) on energy expenditure (A). Following 35 days treatment, mice were placed in CLAMS metabolic chambers for 24 h (12 h dark period as shown by the black bar) and energy expenditure calculated using RER with the following equation: (3.815 + 1.232 x RER) x VO_2_. Average energy expenditure (B), energy expenditure in the light (C) and dark cycles (D) are also included. Values represent the mean ± S.E.M. (n = 6) where *p<0.05 and **p<0.01 is compared to high-fat fed saline treated mice and ^**▽▽▽**^p<0.01 is compared to normal mice.

**Fig 9:**
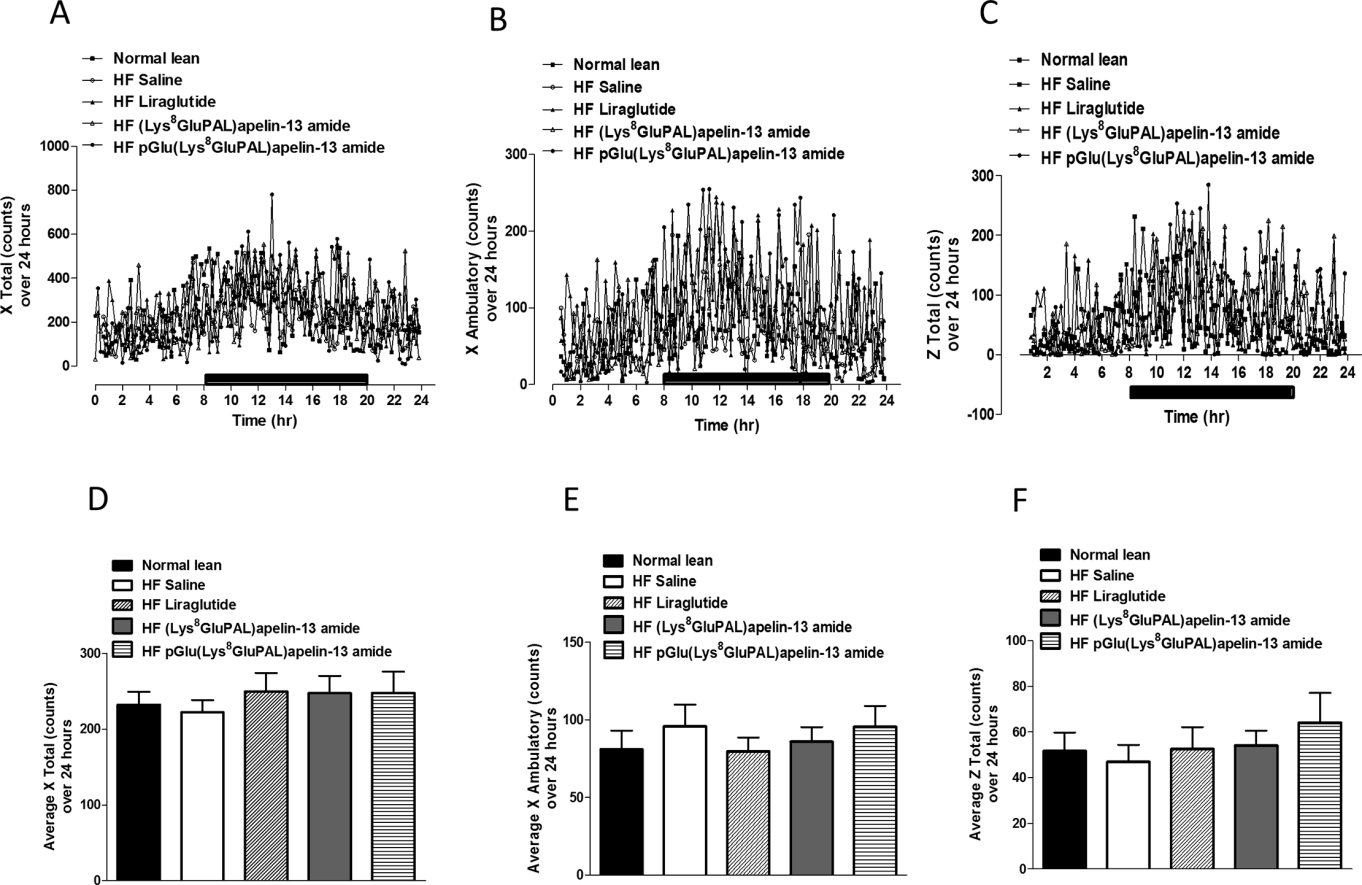
Effect of once daily i.p. administration of liraglutide, (Lys^8^GluPAL)apelin-13 amide or pGlu(Lys^8^GluPAL)apelin-13 amide (each at 25 nmol/kg bw) on locomotor activity. Following 35 days treatment, mice were placed in CLAMS metabolic chambers for 24 h (12 h dark period as shown by the black bar). Activity counts in X-axis (lateral) (A-D) and Z-axis (vertical) (E-F) were recorded every minute for the duration. Values represent the mean ± S.E.M. (n = 6).

**Fig 10:**
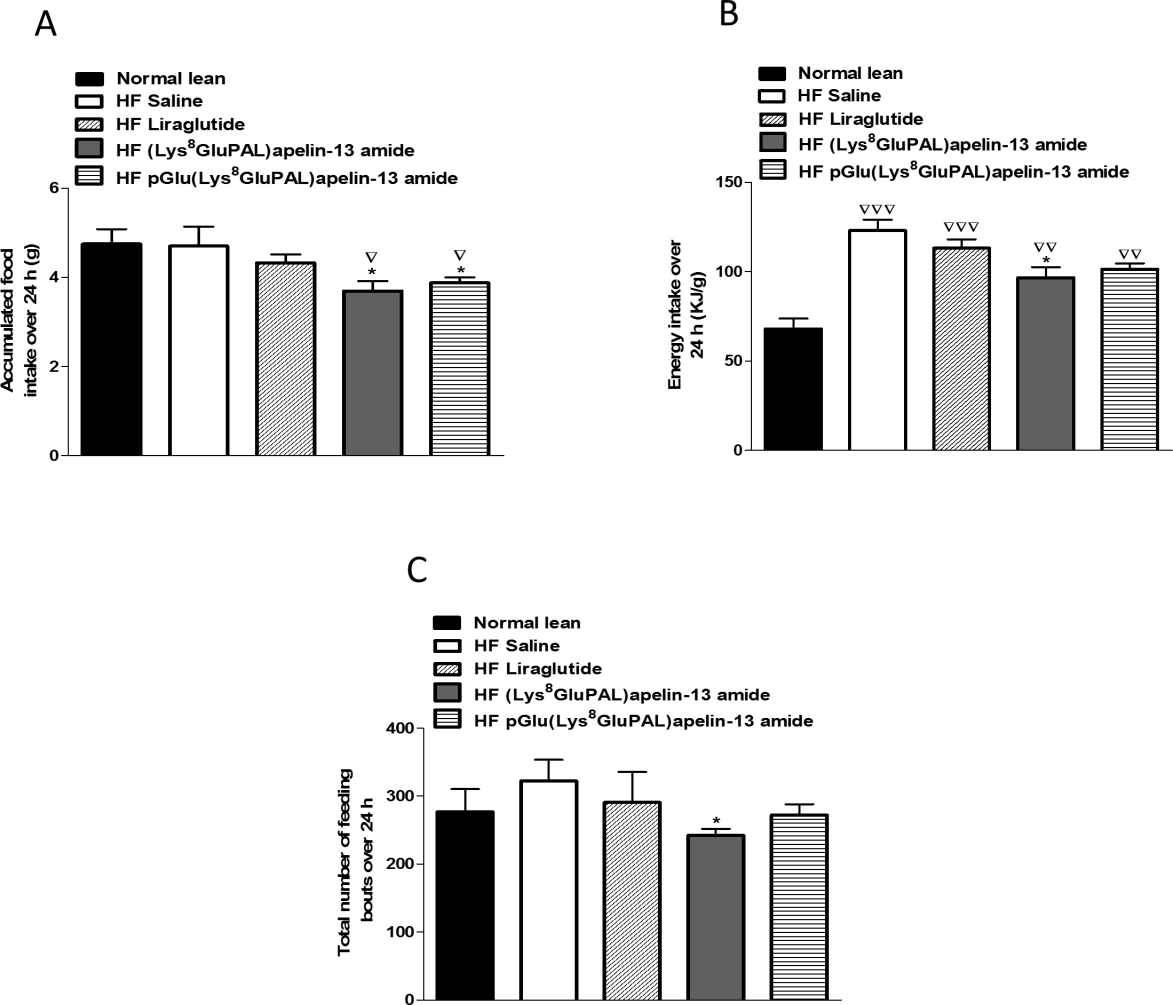
Effect of once daily i.p. administration of liraglutide, (Lys^8^GluPAL)apelin-13 amide or pGlu(Lys^8^GluPAL)apelin-13 amide (each at 25 nmol/kg bw) on food intake. Following 35 days of treatment, mice were placed in CLAMS metabolic chambers for 24 h and food intake (A), energy intake (B) and feeding bouts (C) were measured for the duration. Values represent the mean ± S.E.M. (n = 6) where *p<0.05 compared to high-fat fed saline treated mice and ^**▽**^p<0.05, ^**▽▽**^p<0.01 and ^**▽▽▽**^p<0.001 is compared to mice fed on a normal diet.

Overall oxygen consumption (VO_2_) in high-fat fed mice were unaffected by any of the three peptide treatments (Fig 6A and 6B). Whereas carbon dioxide production VCO_2_ was significantly increased (p<0.05; Fig 6C and 6D) by all three treatments. Liraglutide (p<0.01), and both acylated apelin-13 analogues (p<0.05) also significantly increased average respiratory exchange ratio (RER; Fig 7A and 7B) due to higher activity overall (Fig 7B) as well as during the dark cycle (Fig 7D) but not the light cycle (Fig 7C). These peptide treatments also increased energy expenditure (p<0.05; Fig 8A and 8B) evident from changes during both the light and dark cycles (p<0.05; Fig 8C and 8D). In general, the apelin analogues were more effective at promoting increased energy expenditure than liraglutide when compared against the high fat fed saline treated controls (Fig 8B and 8D). The effects on energy metabolism should be beneficial for weight loss if they can be translated in the human situation. No significant effects were observed in the locomotor activity of any of the peptide treated mice when compared to normal mice of saline treated high fat controls (Fig 9A–9F). In contrast to liraglutide, both groups of apelin treated mice exhibited decreased (p<0.05) cumulative food and energy intake over 24 h (Fig 10A and 10B). In the case of the acylated analogue (Lys^8^GluPAL)apelin-13 amide this was accompanied by decreased energy intake (p<0.05) and a significantly lower number of feeding bouts (p<0.05; Fig 10C).

## 4. Discussion

Numerous studies indicate an emerging involvement of apelin in energy metabolism and the pathophysiology of obesity [39–42]. Both apelin and APJ receptors are present in many tissues including mouse, human and rat adipose tissue and pancreatic islets [20;34,43,44]. Circulating apelin concentrations are increased in obese humans and rodent models of obesity only when accompanied by hyperinsulinaemia [20,45]. This indicates that obesity or high-fat feeding may not be the main cause of elevated apelin, and implies that a close relationship exists between apelin and both the secretion and action of insulin. This highlights possible values of apelin/APJ interactions as an intriguing therapeutic target for obesity and diabetes.

Consistent with this view, recent work in our laboratory has shown that therapeutic natural and stable analogues of apelin-13 stimulate insulin secretion, enhance cellular glucose uptake and improve acute glucose tolerance in animal models of obesity-diabetes [27,34]. Native apelin undergoes extensive enzymatic degradation and rapid plasma clearance *in vivo*. However structural modifications by addition of fatty acid, amide group and/or N-terminal pyroglutamate residue, resulted in bioactive analogues that displayed an enzyme resistant and greatly extended plasma half-life with greater duration of antihyperglycemic actions [28,30,34].

In the present study, examining the antidiabetic potential in high fat fed mice, we chose to used second generation analogues, namely (Lys^8^gluPAL)apelin-13 amide and pGlu(Lys^8^gluPAL)apelin-13 amide. Once daily administration of these peptides for 28 days was associated with robust insulin secretory actions resulting in decreased blood glucose and reduced glucose concentrations, reduced glycemic excursions in in responses to intraperitoneal or oral glucose tolerance studies and elevated pancreatic insulin content. This was accompanied by significantly reduced levels of HbA_1c_. The reduction of may also reflect improvement in insulin action as evidenced by enhanced hypoglycemic action of exogenous insulin. An increase of glucose uptake by skeletal muscle and adipose tissue seems likely [34,46]. Indeed, both (Lys^8^gluPAL)apelin-13 amide and pGlu(Lys^8^gluPAL)apelin-13 amide have been shown to significantly enhance glucose uptake by 3T3-L1 adipocytes *in vitro* [30] corroborating these findings (data not shown). Consistent with this apelin increased glucose uptake, both *in vitro* [30,47] and *in vivo*, through both insulin-dependent and independent pathways [46].

In addition to effects on glucose homeostasis (Lys^8^gluPAL)apelin-13 amide and pGlu(Lys^8^gluPAL)apelin-13 amide significantly reduced food intake and evoked significant body weight loss. These actions together with direct effects discussed above, would also be expected to contribute to the improvements of insulin sensitivity and metabolic control. Further studies using pair-feeding might help assess the relative importance of body weight loss but are difficult to interpret due to effects of ‘meal feeding’ on the parameters under investigation. Apelin and its APJ receptors have been detected in the arcuate and paraventricular nuclei of hypothalamus, known to be key sites in central control of feeding behaviour and energy expenditure [34,48]. Apelin could also alter body adiposity independent of food intake by increasing energy expenditure through activation of mitochondrial uncoupling proteins 1 and 3 [49].

It is also notable that as observed previously [34] these analogues of apelin-13 (increased plasma levels of total GLP-1 suggesting that enhanced secretion of GLP-1 plays a role in the enhanced beta-cell function and glucose homeostasis. The peptide may also contribute to the reduction of food intake and body weight loss via central affects or action to reduce gastric emptying [34]. Interestingly, apelin knockout (KO) mice exhibit reduced insulin sensitivity, glucose intolerance and hyperinsulinaemia [50]. Apelin administration improved insulin sensitivity in these mice with intact APJ receptors [50], with the insulin-sensitising effects continuing for up to 4 weeks, and no evidence of receptor desensitisation.

In high fat fed mice pGlu(Lys^8^gluPAL)apelin-13 amide reduced total cholesterol in high-fat fed mice. Both the fatty acid apelin analogues had positive effect on reducing circulating triglycerides, LDL-cholesterol as well as increasing HDL-cholesterol. Cardiovascular benefits of apelin, including reduction of blood pressure are well established [51] and further studies would be worthwhile to explore such actions in the present context. Once daily administration of the GLP-1 mimetic, liraglutide replicated all of the benefits of apelin-13 analogues but it failed to completely improve lipid profile in high-fat fed mice as shown previously in our lab [38]. Moreover, increased levels of circulating amylase were observed compared to both lean and high-fat fed control, suggesting adverse reactions indicative of pancreatitis [52].

All treated groups including those receiving liraglutide showed increased consumption of O_2_ and production of CO_2_, concomitant with increased energy expenditure. This effect on whole body metabolism was associated with reduction of fat mass. Average respiratory exchange ratio was increased in all groups partly reflecting fat depletion as shown previously with apelin analogues [34]. Apelin analogues were superior to liraglutide in improving bone mineral density, thus negating deteriorating effect of fat mass and body weight. Interestingly, fatty acid apelin analogues showed no changes in ambulatory activity in treated mice suggesting weight loss was independent of activity.

## Conclusion

In conclusion, the present study has shown that once daily administration of (Lys^8^gluPAL)apelin-13 amide or pGlu(Lys^8^gluPAL)apelin-13 amide ameliorated diabetes, evoked weight loss and decreased circulating lipids in high-fat fed mice, with effects similar to or better than liraglutide. Overall the pGlu(Lys^8^gluPAL)apelin-13 amide analogue was the most effective analogue and was better than the non-acylated analogue tested previously [34].

## Abbreviations

APJ: apelin receptor
AUC: integrated area under the curve
BMC: bone mineral content
DEXA: Dual-energy X-ray absorptiometry
GLP-1: glucagon-like peptide-1
pGlu: pyroglutamyl
T2DM: type 2 diabetes mellitus
